# Homogeneous Electrochemical Aptasensor for Sensitive Detection of Zearalenone Using Nanocomposite Probe and Silica Nanochannel Film

**DOI:** 10.3390/molecules28217241

**Published:** 2023-10-24

**Authors:** Zhongnan Huang, Xuan Luo, Fei Yan, Bo Zhou

**Affiliations:** 1 Collaborative Innovation Centre of Regenerative Medicine and Medical Bioresource Development, Application Co-Constructed by the Province and Ministry, Guangxi Medical University, Nanning 530021, China; huangzhongnan@sr.gxmu.edu.cn; 2 Key Laboratory of Surface & Interface Science of Polymer Materials of Zhejiang Province, Department of Chemistry, School of Chemistry and Chemical Engineering, Zhejiang Sci-Tech University, Hangzhou 310018, China; 202020104138@mails.zstu.edu.cn

**Keywords:** zearalenone, homogeneous aptasensor, graphene oxide, vertically-ordered mesoporous silica films

## Abstract

Developing rapid and efficient analytical methods is of great importance for food safety Herein, we present a novel homogeneous electrochemical aptasensor for ultrasensitive quantitative determination of zearalenone (ZEN) based on a nanocomposite probe and silica nanochannel film. X-ray photoelectron spectroscopy, Fourier transform infrared spectroscopy and UV–Vis characterization techniques confirm that graphene oxide (GO) bears an aromatic conjugated structure, along with hydroxyl and carboxyl groups, facilitating the subsequent adsorption of cationic redox hexa-ammine-ruthenium (III) (Ru(NH_3_)_6_^3+^) and anionic ZEN aptamer, to form a Ru(NH3)63+–ZEN aptamer–GO nanocomposite probe in a homogeneous solution. Vertically-ordered mesoporous silica films (VMSF) bearing silanol groups can be simply grown on the solid indium tin oxide (ITO) electrode surface and enable the selective preconcentration of Ru(NH_3_)_6_^3+^, eventually leading to signal amplification. Since the detachment of Ru(NH_3_)_6_^3+^ from the GO surface by the recognized ZEN aptamer in the presence of ZEN, more free Ru(NH_3_)_6_^3+^ is released in solution and produces enhanced redox signals at the VMSF modified ITO electrode, allowing quantitative detection of ZEN. On the basis of the above sensing strategy, the proposed homogeneity, due to the assistance of graphene, as well as of the signal amplification and anti-fouling effects of VMSF, accurate analysis of ZEN can be realized in maize and Chinese chestnut samples.

## 1. Introduction

Mycotoxins produced by fungal species are toxic and often contaminate nearly 25% of the world’s agricultural crops (e.g., wheat, corn and rice), leading to an annual loss of ~5 billion dollars [1]. Zearalenone (ZEN) is one of the most frequent mycotoxins and has severe effects on human health, including teratogenicity, abortion, liver toxicity, blood toxicity, and carcinogenicity [2]. ZEN, with high water insolubility and thermal stability (at 120 °C for 4 h, the toxicity cannot decrease), is difficult to be removed by conventional processing methods, which aggravate its contamination [3]. The International Agency for Research on Cancer has classified ZEN as a class III carcinogen [4]. In addition, the European Union sets the maximum residue levels of ZEN in human food at 75 μg/kg [5]. Due to this low permissible limit and harmfulness, rapid and efficient analytical methods for ZEN determination should be developed for food safety. To date, a great number of techniques have been utilized to detect ZEN, such as lateral flow immunoassays [6], liquid chromatography−tandem mass spectrometry [7], high performance liquid chromatography [8], enzyme-linked immunosorbent assay [9] and spectrometry [10]. These methods possess good accuracy but need complex processing steps, expensive instruments and technical staff. Electrochemical biosensors have received wide attention due to the benefits of convenience, easy miniaturization, good sensitivity, and economy, which have been employed to sensitively determine ZEN [11,12].

Electrochemical biosensors have received growing attraction due to their high specificity and sensitivity, rapidity, and ease of miniaturization and portability [13,14,15,16]. According to the category of biological recognition elements, electrochemical biosensors mainly fall into three kinds: electrochemical immunosensors, enzymatic biosensors and aptamer-based biosensors [17,18,19,20]. By contrast, aptamer-based biosensors are predominant and have been demonstrated to be useful for analytical applications due to their high specificity, chemical and thermal stability, inexpensiveness and successful applicability for diagnosis [21,22,23,24]. In comparison with most reported electrochemical aptasensors using the immobilized aptamer on the electrode surface, homogeneous electrochemical aptasensors are time-saving, free of immobilization steps and suitable for the formation of aptamer-target conjugations [25]. In this situation, all biochemical reactions occur in a homogeneous solution and an electrochemical signal of an indicator for quantification could be detected by the electrode. To improve the analytical sensitivity, nanostructured structures including graphene, metal nanoparticles and nano-porous materials with excellent perma-selectivity, have been applied to promote the electron transfer or the diffusion of indicator to the electrode surface [26,27,28]. Moreover, traditional homogeneous biosensors often require sandwich immunoassays to capture target molecules and probe-labeled antibodies, followed by magnetic separation using immunomagnetic beads before detection. This process involves multiple steps of incubation and washing. Therefore, integration of the amplification effect of nanomaterials with the high affinity of aptamers for the development of novel electrochemical aptasensors with sensitivity and simplicity is expected to bring about substantial advantages for ZEN determination, accompanied by ultra-trace analyses.

Porous materials possess a high specific surface area, adjustable structure and pore size, and excellent mass transfer ability, making them ideal matrices for integrating functional nanomaterials and developing high-performance sensing systems [29,30,31]. Recently, vertically-ordered mesoporous silica films (termed VMSF) with numerous tiny channels and tailored surface charges offer a confined space for gated or selective molecular transport, and have been widely employed for the development of novel electroanalytical sensors based on molecular size, charge, hydrogen bond and lipophilicity [32,33,34,35]. Thanks to the great number of silanol groups existing on the silica channel walls, VMSF shows a pronounced electrostatic perma-selectivity towards cationic species within ultrasmall channels at an appropriate pH, yielding significantly enhanced signals [36,37]. With this enhancement effect, excellent analytical performances in terms of increased sensitivity and decreased detection limit, as well as reduction in the amounts used of expensive signal molecules (e.g., tris(2,20-bipyridyl)ruthenium(II)) [38,39], can be achieved. Herein, we demonstrate a universal and simple homogeneous electrochemical aptasensor with inherent separation capability for sensitive quantitative analysis of ZEN, with the assistance of graphene oxide (GO) and VMSF. Two-dimensional GO nanosheets in a homogeneous solution primarily adsorb cationic Ru(NH_3_)_6_^3+^ and anionic ZEN aptamer to form a Ru(NH_3_)_6_^3+^–ZEN aptamer–GO nanocomposite probe through electrostatic and π-π interactions, which can function as the specifically recognitive and signal element. VMSF with a negatively charged surface grown on the indium tin oxide (ITO) surface (termed the VMSF/ITO electrode) is capable of accelerating mass transport of the free hexa-ammine-ruthenium (III) (Ru(NH_3_)_6_^3+^) electrochemical probe, with positive charge in solution, to the underlying ITO surface, generating amplified redox currents and greatly increasing sensitivity. Target ZEN could bind with ZEN aptamer and pull out Ru(NH_3_)_6_^3+^ from the Ru(NH_3_)_6_^3+^–ZEN aptamer–GO nanocomposite probe, resulting in enhanced redox currents at the VMSF/ITO electrode and enabling the quantitative analysis of ZEN. Compared to traditional homogeneous electrochemical aptasensors, our proposed sensing strategy based on the large Ru(NH_3_)_6_^3+^–ZEN aptamer–GO nanocomposite probe can effectively reduce background signals through size exclusion and thereby eliminate the need for probe separation during detection. Furthermore, accurate quantification of ZEN in food samples (maize and Chinese chestnut) is realized by our simple homogeneous electrochemical strategy.

## 2. Results and Discussion

### 2.1. Principle of the Designed Electrochemical Aptasensor for ZEN Determination

**Scheme 1:**
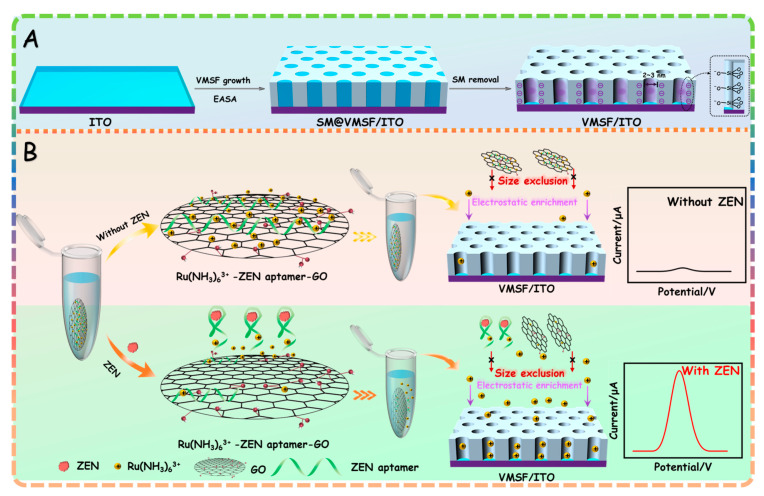
(**A**) Scheme of VMSF/ITO electrode prepared by using EASA method. (**B**) Schematic illustration for the homogeneous electrochemical aptasensor for the determination of ZEN.

Growth of VMSF with an open channel on the ITO surface (termed VMSF/ITO) is performed by the previously reported electrochemically assisted self-assembly (EASA) approach and further exclusion of templated surfactant micelles (SM) from the silica channels (Scheme 1A). VMSF bearing silanol groups could provide numerous sites for the cationic probe in the buffer solution via the electrostatic effect, finally resulting in amplified signals and greatly improving the detection performance. Scheme 1B presents the principle of the proposed homogeneous electrochemical aptasensor by coupling the Ru(NH_3_)_6_^3+^–ZEN aptamer–graphene oxide (GO)–nanocomposite probe and signal amplification of VMSF for Ru(NH_3_)_6_^3+^. As shown, the Ru(NH_3_)_6_^3+^ (signal probe) and ZEN aptamer (recognitive element) are first adsorbed onto the GO nanosheets through electrostatic and π–π interactions, in order to form the Ru(NH3)63+–ZEN aptamer–GO nanocomposite probe. In the absence of ZEN, ZEN aptamer is adsorbed on the GO surface in the random coil state, leading to lesser amount of Ru(NH_3_)_6_^3+^ in solution and generating the relatively low redox signals at the VMSF/ITO electrode. In the presence of ZEN, ZEN aptamer is able to specifically bind with ZEN, which could be pulled out from the GO surface due to the high affinity. In this case, Ru(NH_3_)_6_^3+^ adsorbed on the GO surface is released to the homogeneous solution and displays an enhanced redox signal at the VMSF/ITO electrode. Such redox current variation of Ru(NH_3_)_6_^3+^ generated with the help of GO could be accurately measured by VMSF/ITO electrode, allowing the quantitative determination of ZEN. This newly developed homogeneous electrochemical aptasensor is simple, without immobilization and labeling operations, offering a universal platform for the detection of various analytes by tailoring the suitable aptamer.

### 2.2. Characterizations of GO and VMSF/ITO Electrode

**Figure 1:**
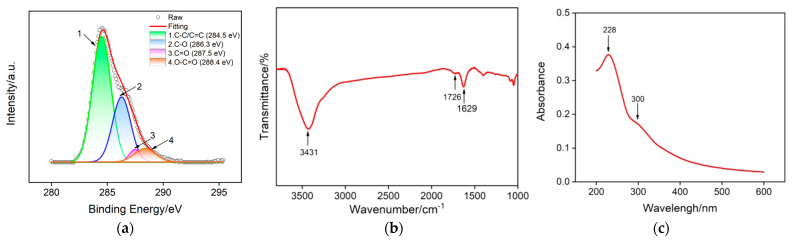
Structure characterization of GO nanosheets: (**a**) high-resolution C 1 s XPS, (**b**) FTIR and (**c**) UV–Vis absorption spectra.

Graphene based nanomaterials with different dimensional structures (e.g., 0D quantum dots [40,41,42,43], 2D sheets [44], and 3D porous foam [45,46,47]) have been widely used in the construction of various novel chemo-/biosensors due to their unique structure and exceptional physico-chemical properties [48,49,50]. Among them, GO nanosheets have a high specific surface area and oxygen-containing groups on their basal plane and edges, which offer plentiful effective binding sites for the electrostatic adsorption of cationic species. Figure 1 shows the high-resolution carbon 1 s X-ray ray photoelectron spectroscopy (XPS), Fourier transform infrared spectroscopy (FTIR) and UV–Vis spectra of GO. As presented in Figure 1a, four obvious characteristic peaks could be found in the carbon 1 s XPS profile of GO. The peak centered at 284.5 eV is attributed to the C-C/C=C of the aromatic rings, and the other three peaks located at 286.3 eV, 287.5 eV and 288.4 eV are assigned to the C-O, C=O and O-C=O, respectively, confirming the existence of oxygen-containing groups in GO nanosheets. Three absorption bands for hydroxyl group peak (3431 cm^−1^), carbonyl group peak (1629 cm^−1^) and carboxyl group (1726 cm^−1^) are found in the FTIR spectra of GO (Figure 1b), further proving the presence of oxygen-containing groups in GO. The UV–Vis spectrum of GO displayed in Figure 1c contains an adsorption peak at 228 nm corresponding to the π→π* transitions of the aromatic C-C bond and a shoulder peak at 300 nm corresponding to the n→π* transition of the C=O bond. All the above results are similar to previous reports and suggest the successful preparation of GO. Moreover, GO bearing an aromatic conjugated structure, hydroxyl and carboxyl groups facilitates the adsorption of cationic species with or without a π-conjugated structure.

**Figure 2:**
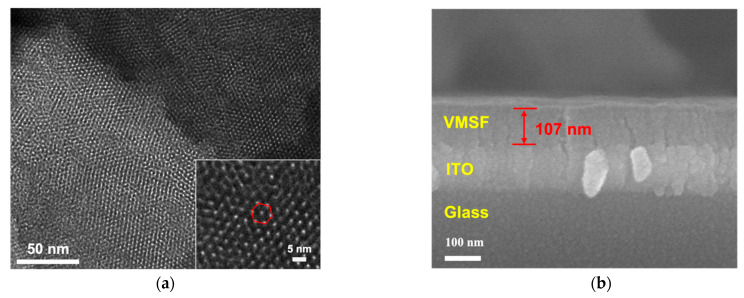

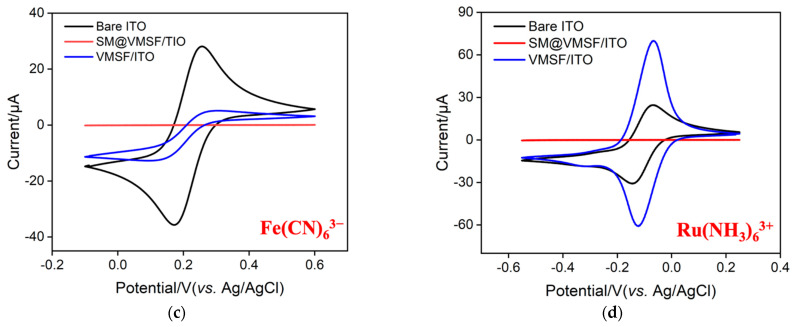
(**a**) Top-view TEM micrographs of VMSF/ITO with various magnified views. The inset shows the hexagonally aligned nanopores. (**b**) SEM micrograph of VMSF/ITO displaying the cross-sectional topography. Cyclic voltammetry (CV) for the bare ITO, and the ITO decorated with SM@VMSF and VMSF recorded in the presence of 0.5 mM (**c**) Fe(CN)_6_^3−^ and (**d**) Ru(NH_3_)_6_^3+^. Supporting electrolyte: 0.05 M KHP (pH = 7.4) solution.

The morphology of VMSF grown on the ITO surface was characterized by electron microscope characterization. As seen in Figure 2a, plentiful and hexagonally aligned nanopores appear as bright spots and the as-prepared silica nanoporous film is intact without cracks over a large area. The diameter and thickness of VMSF are 2~3 nm and 107 nm, respectively (Figure 2a,b). The VMSF layer on the ITO coated glass surface is very homogeneous. The intactness and electrostatic perma-selectivity of VMSF were examined by electrochemical method and the results can be found in Figure 2c,d. Well-defined reversible redox peaks are shown at the bare ITO electrode, which are assigned to the electrochemical reaction of the Fe(CN)_6_^3−^/Fe(CN)_6_^4−^ couple (Figure 2c) and Ru(NH_3_)_6_^3+^/Ru(NH_3_)_6_^4+^ couple (Figure 2d). As demonstrated, when the binary nanocomposite consisting of surfactant micelles (SM) and silica nanochannels was decorated on the ITO electrode surface, both anionic Fe(CN)_6_^3−^ and cationic Ru(NH_3_)_6_^3+^ could not pass through the hydrophobic microenvironment formed by SM, resulting in the capacitive current at the SM@VMSF/ITO electrode and further suggesting the integrity of VMSF on the ITO electrode surface. After exclusion of SM from the silica nanochannels, enhanced redox currents for Ru(NH_3_)_6_^3+^ and declined redox currents for Fe(CN)_6_^3−^ are shown at the VMSF/ITO electrode compared with that obtained at the bare ITO, which results from the electrostatic interaction between negatively charged silica walls and charged probes in the ultrasmall space. Note that VMSF is rich in silanol groups and can deprotonate to display negative charges. The above CV results imply that VMSF with open channels has excellent charge perma-selectivity and indeed shows the basis for the following homogeneous electrochemical aptasensor.

### 2.3. Feasibility of the Homogeneous Electrochemical Aptasensor

**Figure 3:**
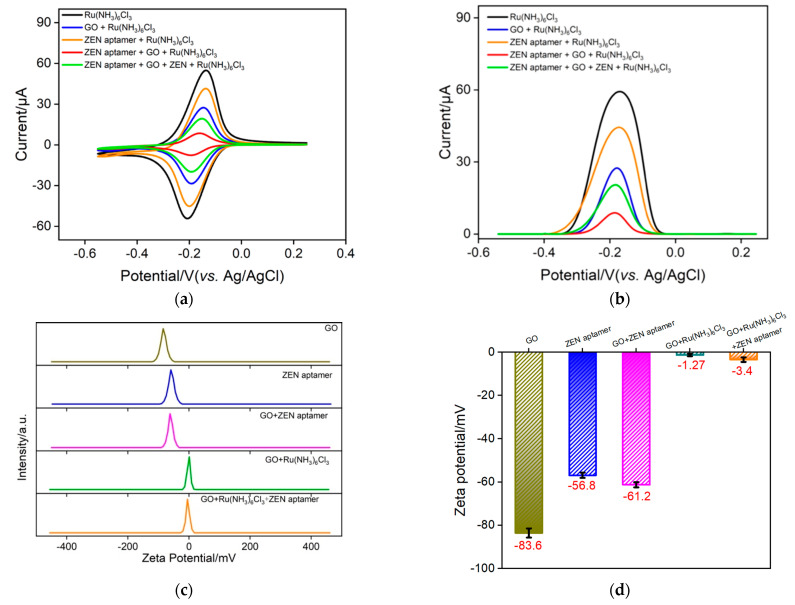
CV (**a**) and DPV (**b**) responses of VMSF/ITO to free Ru(NH_3_)_6_^3+^ in different detected solution. The concentrations of Ru(NH_3_)_6_Cl_3_, ZEN aptamer, GO and ZEN are 100 μM, 1 μΜ, 0.1 mg/mL and 1 ng/mL, respectively. (**c**,**d**) Zeta potential of GO, ZEN aptamer, GO + ZEN aptamer, GO + Ru(NH_3_)_6_Cl_3_ and GO + Ru(NH_3_)_6_Cl_3_ + ZEN aptamer in deionized water. Error bars denote the standard deviations of three measurements.

As illustrated in Scheme 1B, the Ru(NH3)63+–ZEN aptamer–GO nanocomposite electrochemical probe in a homogeneous solution could be employed to specifically recognize ZEN, leading to free Ru(NH_3_)_6_^3+^ and further generating the electrochemical signal. Such redox current variation of Ru(NH_3_)_6_^3+^ is able to be determined by VMSF/ITO owing to the electrostatic amplification effect. To verify this sensing strategy, an electrochemical method, including CV and the corresponding DPV, was first chosen. As revealed in Figure 3a, a pair of well-defined redox peaks due to the electrochemical reactions of Ru(NH_3_)_6_^3+^ is observed at the VMSF/ITO electrode. When GO, ZEN aptamer, or their mixture is added to the solution, the redox signals of Ru(NH_3_)_6_^3+^ dramatically decrease, which results from the electrostatic or π–π interactions between cationic Ru(NH_3_)_6_^3+^ and negatively charged GO/ZEN aptamer, and thereby a smaller amount of free Ru(NH_3_)_6_^3+^ in solution. When target ZEN is present in the solution, Ru(NH_3_)_6_^3+^ could be released from the Ru(NH3)63+–ZEN aptamer–GO nanocomposite probe and finally gives rise to the enhanced redox signals. The corresponding DPV curves are recorded in Figure 3b. In addition, zeta potential is used to confirm the successful formation of the Ru(NH3)63+–ZEN aptamer–GO nanocomposite probe. As revealed in Figure 3c,d, zeta potentials of GO and ZEN aptamer in water are about −83.6 mV and −56.8 mV, exhibiting strong electronegativity. A mixture of GO and ZEN aptamer leads to −61.2 mV, which is comparable to that of ZEN aptamer and indicates the adsorption of ZEN aptamer through the π–π effect. In comparison with GO, ZEN aptamer and their mixture, the presence of cationic Ru(NH_3_)_6_^3+^ could bring about an obvious enhancement in zeta potential, indicating the strong electrostatic adsorption of Ru(NH_3_)_6_^3+^ on the GO nanosheet surface.

### 2.4. Detection of ZEN

**Figure 4:**
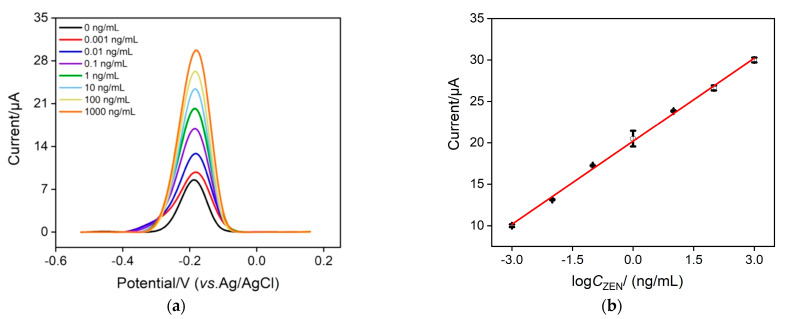
(**a**) DPV curves obtained by our proposed homogenous electrochemical aptasensor in the presence of different concentrations of ZEN (from top to bottom: 0.001, 0.01, 0.1, 1, 10, 100, 1000 ng/mL). (**b**) The linear relationship between the anodic current and the logarithmic value of ZEN concentration. The composite probe solution contains 1 μΜ ZEN aptamer, 0.1 mg/mL GO and 100 μΜ Ru(NH_3_)_6_Cl_3_. Error bars denote the standard deviations of three measurements.

As Ru(NH_3_)_6_^3+^ is chosen as the signal indicator, we investigate the optimal concentration of Ru(NH_3_)_6_^3+^ to achieve the best analytical performance. Figure S1 records the anodic peak currents (left) and change of anodic peak current (Δ*I* = *I* − *I*_0_) (right) of the Ru(NH3)63+–ZEN aptamer–GO nanocomposite probe with (*I*) and without (*I*_0_) 10 ng/mL ZEN at various concentrations of Ru(NH_3_)_6_^3+^. As displayed, Δ*I* increases with the increasing concentration of Ru(NH_3_)_6_^3+^ and reaches optimal value at a concentration of 100 μΜ, which is thereby selected as the best condition in this study. Under optimal experimental conditions, DPV curves at various concentrations of ZEN were recorded using our proposed homogeneous electrochemical aptasensor. As depicted in Figure 4a, when the ZEN concentration changes from 1 pg/mL to 1 μg/mL, the anodic current obtained at the VMSF/ITO electrode gradually enhances with increasing ZEN concentration. The magnitude of the anodic current is proportional to the logarithmic concentration of ZEN (Figure 4b), generating a well-fitting linear equation of *I* (μA) = 3.34 log*C*_ZEN_ + 20.2 (*R*^2^ = 0.998) and a low limit of detection (LOD) of 1.2 fg/mL (Signal/Noise = 3). By comparing the sensing performance of our proposed homogeneous strategy with other sensing approaches, a better linear dynamic range and a lower LOD are shown in Table 1. Moreover, most sensing methods reported previously involve complex labeling steps, which could be efficiently simplified by our proposed label-free homogeneous electrochemical sensor.

Starch, ochratoxin (OTA), and aflatoxin (AFB_1_) were used to examine the selectivity of the proposed homogeneous electrochemical aptasensor combined VMSF/ITO and nanocomposite. Figure S2 shows the variation of anodic peak current response (*I* − *I*_0_) to the ZEN after the addition of interfering species. It could be found that the quantity of starch, OTA and AFB_1_ is remarkably smaller than that of ZEN or a mixture containing ZEN, suggesting the satisfactory selectivity of our proposed homogenous electrochemical sensing strategy. The reproducibility of the proposed homogeneous electrochemical aptasensor was studied by testing a known concentration of ZEN using five different VMSF/ITO electrodes. The relative standard deviation for five measured electrochemical signals is 3.7%, showing appreciable reproducibility of the proposed sensing strategy.

### 2.5. Analytical Application in Food Samples

To examine the feasibility of our designed homogeneous electrochemical aptasensor in practical application, maize and chestnut samples were employed. By mixing 5 g maize or chestnut powder with 20 mL acetonitrile solution of 8.5 mM NaCl under ultrasonication and centrifuging at 6000 rpm for 10 min, supernatant was achieved, followed by dilution using buffer solution (0.01 M PBS (pH = 7.4)). Thus, food samples for measurement were obtained. Various standard amounts of ZEN were artificially added into the above food samples and determined by the proposed homogeneous electrochemical aptasensor. As implied in Table 2, satisfactory recoveries in the range of 96.0–105% and low RSD value (less than 2.1%) were obtained, demonstrating the accuracy and successful applicability of the developed homogeneous electrochemical sensing strategy.

## 3. Materials and Methods

### 3.1. Chemicals and Materials 

Graphene oxide (GO) was prepared by a modified Hummers method [58]. Sodium dihydrogen phosphate dehydrate (NaH_2_PO_4_), sodium phosphate dibasic dodecahydrate (Na_2_HPO_4_), potassium ferricyanide (K_3_[Fe(CN)_6_]), potassium ferrocyanide (K_4_[Fe(CN)_6_]), tetraethyl orthosilicate (TEOS) and hexadecyl trimethyl ammonium bromide (CTAB) were purchased from Aladdin (Shanghai, China). Hexaammineruthenium (III) chloride (Ru(NH_3_)_6_Cl_3_) was bought from Sigma-Aldrich (Shanghai, China). Ethanol (EtOH) and sodium nitrate (NaNO_3_) were obtained from Hangzhou Gaojing Chemistry Co., Ltd. (Hangzhou, China). The ZEN aptamer (Apt) 5′-TCATCTATCTATGGTACATTACTATCTGTAATGTGATATG-3′ was synthesized by Sangon Biotech. Co., Ltd. (Shanghai, China). Aflatoxin (AFB_1_), ochratoxin (OTA) and zearalenone (ZEN) were provided by the College of Food Science, Zhejiang University (Hangzhou, China). Maize and chestnut were obtained from the local supermarket (Hangzhou, China). ITO coated glasses (<17 Ω/square, thickness: 100 ± 20 nm) were obtained from Zhuhai Kaivo Optoelectronic Technology, which were first cleaned using 1 M NaOH aqueous solution, and then sonicated in acetone, ethanol, and deionized water, respectively. Ultrapure water (18.2 MΩ cm) was used to prepare all aqueous solutions throughout this work.

### 3.2. Apparatus

A field-emission scanning electron microscope (SEM, S-4800, Hitachi, Tokyo, Japan) and a transmission electron microscope (TEM, JEM-2100, JEOL Ltd., Musashino, Japan) were used to investigate the morphology of VMSF. The acceleration voltages for SEM and TEM are 5 kV and 100 kV, respectively. Ultraviolet-Vis (UV–Vis) absorption spectra were performed on a UV–Vis spectrometer (UV-2450; Shimadzu, Kyoto, Japan). X-ray photoelectron spectroscopy (XPS) analysis (250 W, 14 kV, Mg Kα radiation) was carried out on a PHI5300 electron spectrometer (PE Ltd., Waltham, MA, USA). A Vertex 70 spectrometer (Bruker, Woodlands, TX, USA) was used to measure the Fourier transform infrared spectroscopy (FTIR) data through the KBr tablet method. The zeta potential characterization was taken on a SZ-100V2 nano particle analyzer (HORIBA, Tokyo, Japan) with a He-Ne laser (633 nm) at 90 angle collecting optics at 25 °C. Cyclic voltammetry (CV) and differential pulse voltammetry (DPV) measurements were performed an Autolab PGSTAT302N electrochemical workstation (Metrohm, Herisau, Switzerland) with a three-electrode system consisting of bare or modified ITO as the working electrode, Ag/AgCl (saturated KCl solution) as the reference electrode and Pt wire electrode as the auxiliary electrode. The parameters for DPV tests are step (0.005 V), modulation amplitude (0.05 V), modulation time (0.05 s) and interval time (0.2 s).

### 3.3. Preparation of the VMSF/ITO

VMSF was grown on the cleaned ITO electrode (1 cm × 0.5 cm) by EASA method according to the previous reports [59]. Specially, 3.050 mL of TEOS and 1.585 g of CTAB were added to the mixture consisting of 20 mL of NaNO_3_ aqueous solution (0.1 M, pH = 2.6) and 20 mL of ethanol. Then the above solution required pre-hydrolyzation under stirring for 2.5 h to obtain the silica-based precursor. The bare ITO electrode underwent a cleaning procedure and was soaked in the above mixture under quiescent conditions along with the reference electrode (Ag/AgCl) and auxiliary electrode (Pt sheet). Experimental conditions for the growth of VMSF: a constant current density, −0.70 mA·cm^−2^, 10 s. Finally, the growth of VMSF was terminated immediately by rinsing with amounts of ultrapure water and the obtained modified electrode was further aged at 120 ^◦^C overnight. The modified electrode, keeping surfactant micelles (SMs) inside the nanochannels, was denoted as SM@VMSF/ITO. After exclusion of SMs by treatment of SM@VMSF/ITO electrode with 0.1 M HCl–ethanol solution under stirring for 5 min, VMSF with open channels on the ITO surface was achieved, named VMSF/ITO.

### 3.4. Preparation of the Ru(NH3)63+–ZEN Aptamer–GO Nanocomposite Probe and Detection of ZEN

10 μL of 10 mg/mL GO, 10 mM Ru(NH_3_)_6_Cl_3_ and 100 μM ZEN aptamer were mixed in a 1 mL 0.01 M PBS (pH = 7.4) solution and incubated at 37 °C for 2 h, to obtain the Ru(NH3)63+–ZEN aptamer–GO nanocomposite probe. After mixing various concentrations of ZEN with the Ru(NH3)63+–ZEN aptamer–GO nanocomposite probe and further incubating at 37 °C for 1 h, the VMSF/ITO electrode was used to measure the redox current variation of Ru(NH_3_)_6_^3+^, allowing the quantitative detection of ZEN. 

## 4. Conclusions

We demonstrated a simple homogeneous electrochemical aptasensor for ultrasensitive determination of ZEN in maize and Chinese chestnut samples in a combination of the assistance of GO and signal amplification of VMSF. The Ru(NH3)63+–ZEN aptamer–GO nanocomposite probe was formed in a homogeneous solution through electrostatic and π–π interactions, which could serve as the recognitive and signal element. When ZEN was present, ZEN aptamer was specifically dissociated from the nanocomposite probe and further triggered the release of Ru(NH_3_)_6_^3+^, which could be determined by VMSF/ITO electrode and generate the increased redox currents. The VMSF could electrostatically preconcentrate the cationic Ru(NH_3_)_6_^3+^ probe and captured the variation of Ru(NH_3_)_6_^3+^ amounts in solution, enabling highly sensitive determination of ZEN. This sensing strategy could not only avoid the time-consuming complicated process of electrode modification and labeling, but also provide a highly sensitive and universal approach for a variety of analytes.

## Figures and Tables

**Table 1:** Comparison of linear range and LOD of the proposed homogeneous electrochemical aptasensor with previous sensing methods for the determination of ZEN.

Electrode	Detection Method	Linear Range (ng/mL)	LOD (ng/mL)	References
-	PL	0.001–50	2.2 × 10^−4^	[51]
-	FL	0.001–10	2.1 × 10^−4^	[52]
-	FL	0.01–100	3.6 × 10^−4^	[53]
-	Colorimetry	20–8 × 10^4^	10	[54]
Au-PANI-Au/GCE	i-t	1 × 10^−6^–10^2^	4.5 × 10^−7^	[55]
AuNPs/Fe_3_O_4_NRs/rGO/AuE	EC	0.5 × 10^−3^–50	1.1 × 10^−4^	[56]
PtNi@Co-MOF/AuE	EC	10 × 10^−6^–10	1.37 × 10^−6^	[57]
homogeneous electrochemical aptasensor	EC	0.001–1000	1.2 × 10^−6^	This work

FL: fluorometry; Au-PANI-Au: Au-Polyaniline-Au nanohybrids; GCE: Glass carbon electrode; AuNPs/Fe_3_O_4_NRs/rGO: Gold nanoparticles Fe_3_O_4_ nanorods/reduced graphene oxide; AuE: Gold electrodes; PtNi@Co-MOF: PtNi@Co-Metal-organic frameworks.

**Table 2:** Analysis of ZEN in maize and chestnut samples.

Sample	Added (ng/mL)	Found (ng/mL)	Recovery (%)	RSD (%)
Maize ^a^	0.0100	0.00971	97.1	1.2
	0.1	0.103	103	2.1
	1000	992	99.2	1.2
Chestnut ^a^	0.0100	0.00960	96.0	1.9
	0.100	0.105	105	1.9
	1000	991	99.1	0.92

^a^ The achieved maize and chestnut samples were first diluted by a factor of 50 times using buffer solution.

## Data Availability

The data presented in this study are available on request from the corresponding author.

## Supplementary Materials

The following supporting information can be downloaded at: https://www.mdpi.com/article/10.3390/molecules28217241/s1, Figure S1: Optimization of experimental conditions; Figure S2: Anti-interference ability of homogeneous electrochemical aptasensor.
