# Supplementary material for: Homogeneous Electrochemical Aptasensor for Sensitive Detection of Zearalenone Using Nanocomposite Probe and Silica Nanochannel Film
Source: Molecules. 2023 Oct 24;28(21):7241. doi: 10.3390/molecules28217241 (PMC10647457; doi:10.3390/molecules28217241)
Supplement: Supplementary file 1 [file molecules-28-07241-s001.zip › molecules-2643428-supplementary.pdf]

Supporting Information to

Homogeneous electrochemical aptasensor based on graphene-assistance and nanochannel amplification for sensitive detection of ZEN in maize and Chinese chestnut samples

Zhongnan Huang [1,†], Xuan Luo [2,†], Fei Yan [2,*] and Bo Zhou [1,*]

[1] Collaborative Innovation Centre of Regenerative Medicine and Medical Bioresource Development, Application Co-Constructed by the Province and Ministry, Guangxi Medical University, Nanning 530021, China

[2] Key Laboratory of Surface & Interface Science of Polymer Materials of Zhejiang Province, Department of Chemistry, School of Chemistry and Chemical Engineering, Zhejiang Sci-Tech University, Hangzhou 310018, China

* Correspondence: yanfei@zstu.edu.cn or feifei19881203@126.com (F.Y.); zhoubo@gxmu.edu.cn (B.Z.)

† These authors contributed equally to this work.

Table of Content

S1. Optimization of experimental conditions

S2. Anti-interference ability of homogeneous electrochemical aptasensor

S1. Optimization of experimental conditions

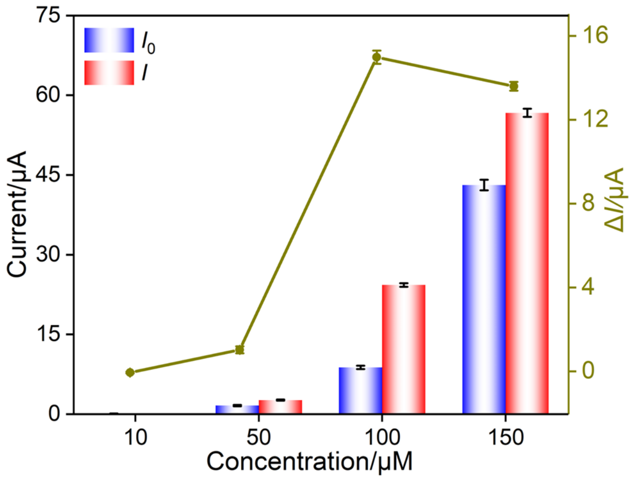

Figure S1 Optimization for the concentration of $Ru(NH_3)_6Cl_3$. Primary axis is the anodic peak current generated from $Ru(NH_3)_6^{3+}$-ZEN aptamer-GO nanocomposite probe in the absence (I_0) and presence (I) of 10 ng/mL ZEN. The nanocomposite probe contains 1 μM ZEN aptamer, 0.1 mg/mL GO and various concentration of $Ru(NH_3)_6Cl_3$ ranging from 10 μM to 150 μM. Secondary axis is the corresponding anodic peak current variation (ΔI) before (I_0) and after (I) incubating 10 ng/mL ZEN with the $Ru(NH_3)_6^{3+}$-ZEN aptamer-GO nanocomposite probe. Error bars denote the standard deviations of three measurements.

S2. Anti-interference ability of homogeneous electrochemical aptasensor

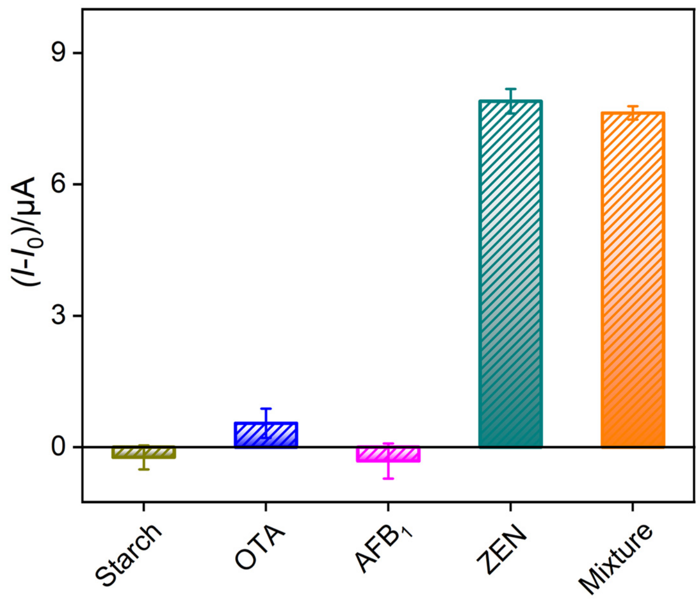

Figure S2 Selectivity of the proposed aptasensor towards ZEN. The concentrations of ZEN and starch are 0.1 ng/mL and 10 μg/mL, respectively. And the concentration of AFB_1 and OTA is 10 ng/mL. Error bars denote the standard deviations of three measurements.
